# The University of California San Diego performance-based skills assessment: a useful tool to detect mild everyday functioning difficulties in HIV-infected patients with very good immunological condition

**DOI:** 10.1007/s13365-020-00891-8

**Published:** 2020-08-24

**Authors:** Valentina Delle Donne, Nicoletta Ciccarelli, Valentina Massaroni, Alberto Borghetti, Alex Dusina, Damiano Farinacci, Elena Visconti, Enrica Tamburrini, Massimiliano Fabbiani, Simona Di Giambenedetto

**Affiliations:** 1 Infectious Diseases Institute, Department of Safety and Bioethics, Catholic University of Sacred Heart, Rome, Italy; 2 Department of Psychology, Catholic University of the Sacred Heart, Largo Agostino Gemelli 1, 20123 Milan, Italy; 3 UOC Infectious Diseases, Fondazione Policlinico Universitario A. Gemelli IRCCS, Rome, Italy; 4 Tropical and Infectious Diseases Unit, Department of Specialized and Internal Medicine, University Hospital of Siena, Siena, Italy

**Keywords:** Everyday functioning, Functional assessment, Cognition evaluation, HIV-associated neurocognitive disorders

## Abstract

Everyday functioning (EF) impairment is frequent in people living with HIV (PLWH). Our aim was to better explore EF and its association with PLWH cognition, by administering both the IADL scale, the most common functional scale, and a new and ecologic multi-domain (communication and financial skills) tool to measure EF as the University of California San Diego **(**UCSD) Performance-Based Skills Assessment-Brief Version (UPSA-B). Eighty-five PLWH on cART with very good immunological condition and 23 age- and education-matched healthy controls (HC) were enrolled. PLWH underwent a standardized neuropsychological battery plus IADL, and cognitive impairment was defined according to Frascati criteria. Both groups underwent the UPSA-B. Only 6 subjects (7%) were affected by cognitive impairment (asymptomatic profile). While IADL score was at ceiling for all patients, the UPSA-B total score was significantly worse in PLWH when compared with HC [mean 82.1 (SD 9.3) vs 89.2 (SD 6.2); *p* < 0.001]. At communication subtest, PLWH group and HC were significantly different (*p* = 0.002), while no difference emerged at financial skills (*p* = 0.096). Higher score at UPSA-B was independently associated with better global cognitive performance (composite Z-score) (*β* 7.79; *p* < 0.001). Also considering each single cognitive domain, UPSA-B performance (both total and at subtests) confirmed the association with neurocognitive performance. In conclusion, UPSA-B seems to better discriminate EF impairment than IADL in PLWH, and it was associated with cognitive functions, also in the absence of symptomatic cognitive impairment. Thus, it appears a promising tool in the context of HIV infection to avoid misdiagnosis and to better detect also mild EF.

## Introduction

HIV is a chronic illness, frequently resulting in everyday functioning (EF) impairment. It is estimated that nearly 60% of people living with HIV (PLWH) infection have problems with EF (Blackstone et al. 2013). EF impairment may affect basic daily activities as cooking and housekeeping (Heaton et al. 2004), driving (Marcotte et al. 1999), and job efficiency (Blackstone et al. 2013; Gorman et al. 2009; Heaton et al. 2004; Scott et al. 2011). The functional damage in everyday life is a notable public health concern, since it is associated to reduce ability to care for oneself and properly manage HIV disease (Scott et al. 2011; Thames et al. 2011).

EF impairments among PLWH may be due to a combination of several factors, and it is still not well understood because of its factorial nature. Some predictors of EF impairment are lower cognitive reserve, neuropsychiatric complications, mood, inadequate motivation, substance abuse, and advanced HIV disease (Blackstone et al. 2013; Heaton et al. 2004; Kamat et al. 2012; Milanini et al. 2016a, 2016b; Sadek et al. 2007). Among others, neurocognitive impairment is a strong and substantial predictor of EF deficits in HIV disease, mainly when involving executive functions, learning, attention, working memory, and linguistic abilities (Heaton et al. 2004).

The introduction of combined antiretroviral therapy (cART) has produced a reduction in the overall incidence of central nervous system (CNS) pathologies and of the most severe forms of HIV-associated neurocognitive disorders (HAND). However, prevalence of HAND is still approximately 25–50% because of milder forms persistence that still affect PLWH despite the antiretroviral therapy (Ciccarelli et al. 2013; Ciccarelli et al. 2019; Fabbiani et al. 2015; Fabbiani et al. 2019; Heaton et al. 2011; Milanini et al. 2016a, 2016b; Sacktor et al. 2016; Simioni et al. 2010).

The current diagnostic categories of HAND, named “Frascati criteria,” recognize three conditions: asymptomatic neurocognitive impairment (ANI), mild neurocognitive disorder (MND), and HIV-associated dementia (HAD) (Antinori et al. 2007; Heaton et al. 2010); the discrimination between the first two patterns depends on functional impairment that must be absent for ANI. Currently, the ANI profile is the most represented in PLWH affected by HAND, especially in those with suppressed plasma viral load (50% of HAND cases, Robertson et al. 2007). Indeed, on the basis of Frascati criteria classification, EF has a central role for the diagnostic differentiation between HAND subcategories (Antinori et al. 2007).

Many tools have been proposed for the assessment of EF impairment in PLWH, each of these showing some limitations. Usually, EF is measured by IADL scale (Instrumental Activities of Daily Living, Lawton and Brody 1969), but this tool has shown low sensitivity in patients with no overt dementia, and, as a self-reported questionnaire, it is sensitive to errors due to retrospective recall, cognitive deficits, state-dependent biases (e.g., depressed mood), affective distress, and level of insight (Heaton et al. 2004). Furthermore, self-reported attributions may be closely related to poor metacognition, often affected by HAND (Casaletto et al. 2014; Juengst et al. 2012), even in case of MND and ANI (Chiao and Blizinsky 2013). Considering these limitations, MND is likely underrated, and a misdiagnosis of asymptomatic HAND could result in delayed interventions, more rapid disease progression, imprecise clinical recommendations, and an increased risk of public health concerns (Obermeit et al. 2017).

Previous studies have highlighted the importance to use an ecologic and multi-domain EF assessment, by adding a performance-based evaluation to the common self-report scales, in order to better differentiate asymptomatic versus symptomatic HAND (Blackstone et al. 2012). In this regard, many American studies had evaluated clinical utility of the University of San Diego (UCSD) Performance-Based Skills Assessment-Brief Version (UPSA-B) in PLWH.

UPSA-B is the short version of a performance-based assessment used to measure functional capacity that expends 10 min and assesses financial and communication skills through 4 tasks: counting money and making change, paying bills, telephone use, and a medical appointment rescheduling task. It was originally developed and validated in samples of individuals with serious mental illness, showing sensitivity in detecting psychiatric disorders and resulting associated with employment status, residential independence, daily living skills, and work skills (Mausbach et al. 2007; Patterson et al. 2001).

Afterwards it was noticed that also patients with “amnestic” mild cognitive impairment performed significantly worse than a cognitively healthy group on the UPSA that showed a good sensitivity in detecting minor functional damage (Goldberg et al. 2010). However, literature still reports inconsistent results in PLWH. In particular, on one hand, UPSA-B was able to differentiate PLWH with HAND from PLWH without HAND (Moore et al. 2017; Sheppard et al. 2018), but on the other hand, it was relatively ineffective to distinguish PLWH from healthy controls when authors controlled for group differences in sex and education (Moore et al. 2017); moreover, the sensitivity and specificity values and the cutoff scores differed across studies, and no associations with manifest functioning variables (e.g., employment status, IADL dependence) or quality of life in PLWH have been found.

Thus, additional studies are needed to consolidate sensitivity of UPSA-B in detecting PLWH affected by milder forms of cognitive and EF impairment. Our aim was to better explore EF and its association with cognitive functioning in an Italian cohort of PLWH, by administering both the UPSA-B and the IADL scale.

## Methods

### Participants

PLWH were consecutively enrolled from October 2018 to June 2019 during routine outpatient visits at Infectious Diseases Institute of “Policlinico Gemelli Foundation” of Rome. Twenty-three age- and education-matched healthy control (HC) were also enrolled.

Exclusion criteria in patients’ selection were as follows: age < 18 years, active/past CNS opportunistic infections, history of neurological disorders, active psychiatric disorders, alcoholism or drug abuse, and difficulties with the Italian language.

Data on the following demographic, clinical, and laboratory variables were collected for each participant at the time of neuropsychological testing: sex, age, education, ethnicity, risk factors for HIV infection, time from HIV diagnosis, current and past antiretroviral regimen, current and nadir CD4 cell count, HIV-1 viral load, co-infection with hepatitis C virus (HCV), comorbidities, and concomitant medications.

HC had no history or risk factors for neurologic impairment and were not taking any medication deemed to affect cognitive abilities. They were recruited among hospital personnel or patients’ caregivers or relatives. All subjects were volunteers. They did not receive any financial remuneration for participating.

The study was approved by the institutional ethic committee and all participants provided written informed consent prior to enrollment.

### EF measures

To measure EF, IADL scale was administered; moreover, both groups underwent UPSA-B assessing financial and communication skills.

- IADL questionnaire (Instrumental Activities of Daily Living) (Lawton and Brody 1969): a self-report measure that asks participants to answer questions relating to the level of assistance needed in managing finances, transportation, using the telephone, cooking, housekeeping and laundry, buying groceries, and responsibility for own medication.
- UCSD Performance-Based Skills Assessment-Brief Version (UPSA-B) (Mausbach et al. 2007): a measure of functional capacity (on a 0–100 scale where higher scores correspond to better EF) in which subjects are asked to role-play tasks in 2 areas of functioning: financial skills and communication skills, which are assessed independently. In the financial skills module, participants were asked to count change, generate change from an item purchased at a store, and pay a utility bill. In the communication skills module, participants were asked to dial a telephone to contact emergency services, call information in order to obtain a telephone number, and call a doctor in order to reschedule an appointment.

### Neuropsychological examination

PLWH underwent a standardized neuropsychological test battery (SNB), exploring memory, attention, language, executive functions, and fine motor abilities. Cognitive areas were investigated as follows:

- Learning memory: the Rey Auditory Verbal Learning Test (RAVLT)
- Attention: WAIS Digit Span, WAIS Digit Symbol
- Verbal fluency: FAS Test (subtest of the Neurosensory Center Comprehensive Examination for Aphasia-NCCEA)
- Executive functioning: multiple features target cancelation (MFTC)
- Fine motor skills: Grooved Pegboard Test

Global performance was measured by transforming raw scores at each task into standardized Z-scores using means and standard deviations of Italian normative data (Capitani and Laiacona 1997; Carlesimo et al. 1996) and averaging to calculate a composite total and for each domain score. Domain-specific impairment was defined as a Z-score less than or equal to − 1. According to Frascati criteria (Antinori et al. 2007), patients were considered affected by cognitive disease if they showed impairment in at least two domains. The profiles of ANI, MND, and HAD were differentiated on the basis of the degree of cognitive and EF impairment (IADL score).

Each patient was also submitted to the following assessments:

- SF-36 (36-item Short-Form Health Survey) (Ware et al. 1993) investigating health-related quality of life, which consists of 2 summary scores: physical health (physical functioning, physical role limitations, bodily pain, general health) and mental health (emotional role limitations, energy/fatigue, social functioning, emotional well-being). Each area ranges from 0 to 100 (mean 50, SD 10), and higher scores correspond to better health status. Elevated negative perception of both mental and physical health was considered if patient scored at least 1 SD below the normative mean.
- Zung Self-Rating Depression Scale (Zung 1965; Zung 1986): a short self-report questionnaire which survey four common characteristics of depression: the pervasive effect, the physiological equivalents, other disturbances, and psychomotor activities. A score ≥ 50 is considered corresponding to probable depression.
- Adherence to therapy questionnaire: a self-report judgment about accuracy in taking antiretroviral drugs on a 0–100 Visual Analogue Scale (VAS) (Murri et al. 2010).

HC underwent a Mini Mental State Examination (MMSE) to provide a brief global measure of cognitive functioning and were enrolled with a score ≥ 27.

### Statistical analysis

Descriptive statistics were calculated for quantitative variables [median, interquartile range (IQR), mean, standard deviation (SD)] and qualitative variables (percent frequencies).

*t* tests were performed to determine group differences in the UPSA-B total scores and for the two sub-domains as finances and communication. Also effect sizes were provided by calculating Cohen’s d (d), in order to show the clinical significance of the UPSA comparisons between PLWH and HC.

We performed multivariate linear regression analyses to explore association between UPSA-B scores and cognitive performance, adjusting for variables showing a *p* value < 0.100 in univariate analysis. A 2-tailed *p* value of less than 0.05 was considered statistically significant. All analyses were performed using the SPSS version 21.0 software package (SPSS Inc., Chicago, IL).

## Results

### Demographic and clinical characteristics

A total of 85 Italian PLWH on cART and 23 HC were enrolled; their demographic and clinical characteristics are summarized in Table 1. Of the 85 PLWH, 6% (*n* = 5) were HCV co-infected and 28% (*n* = 24) with past AIDS-defining events. Median adherence to cART was 95 (IQR 85–100) on a 0–100 VAS scale. Subjects in the two groups were matched for age (*p* = 0.967) and education (*p* = 0.951) but significantly differed for sex (*p* = 0.005).

**Table 1:** Demographic and clinical characteristics of PLWH group and HC

Parameters	PLWH (*n* = 85) *N* (%) or median (IQR)*	HC (*n* = 23) *N* (%) or median (IQR)*	*p*
Male sex	66 (78)	11 (48)	**0.005**
Age (years)*	54 (48–60)	53 (47–58)	0.967
Education (years)*	13 (11–17)	13 (12–17)	0.951
Smokers	10 (12)	–	
Cannabis use	2 (2.4)	–	
Alcohol use	1 (1.2)	–	
Other drug use	1 (1.2)	–	
Transmission risk factor		–	–
Homosexual	35 (44)		
Heterosexual	34 (43)		
Injecting drug users	10 (13)		
Time from HIV diagnosis (years)*	14 (4–24)	–	–
Time from first cART (years)*	12 (4–20)	–	–
Time from current cART (years)*	2 (0.9–2.7)	–	–
HIV-RNA < 50 copies/mL	79 (96)	–	–
CD4 cell count (cell/μL)*	551 (121–790)	–	–
CD4 nadir (cell/μL)*	100 (6–270)	–	–

Bold values represent statistically significant *p* values

*N* number, *IQR* interquartile range, *PLWH* people living with HIV, *HC* healthy control, *cART* combined antiretroviral therapy

*Median and IQR reported

### Neuropsychological evaluation

All PLWH underwent SNB. The total mean Z-score on SNB was 0.36 (SD 0.63) with only 6 subjects (7%) who were affected by HAND, all with the ANI profile according to IADL score that was at ceiling (8/8) for all subjects.

Considering each cognitive domain separately, a higher proportion of impairment was observed in motor abilities (13%), followed by memory (7%), linguistic skills (5%), and attention and executive functions (2%). Results of the neuropsychological performances in each cognitive domain are shown in Table 2.

**Table 2:** Neuropsychological performances in each cognitive domain

Cognitive domains	Mean Z-score (SD)	N (%) of patients with domain-specific impairment^a^
Global Z-score^b^	0.36 (0.63)	4 (4.7%)
Memory	0.39 (1.01)	6 (7%)
Attention	0.54 (0.77)	3 (3.5%)
Executive functions	0.12 (0.57)	2 (2.4%)
Language	0.72 (1.05)	4 (4.7%)
Fine motor skills	0.05 (1.12)	11 (13%)

*N* number, *SD* standard deviation

^a^Based on Italian normative data

^b^Average of single Z-score on each domain

Overall, mean Zung Depression Scale score was 35 (SD 8.3), and 8.2% (*n* = 7) of PLWH show a probable depression. About the health-related quality of life (SF-36), 29.4% (*n* = 25) and 18% (*n* = 16) of the PLWH showed, respectively, an elevated negative perception of mental and physical health.

### Everyday functioning evaluation

Evaluating EF by IADL, scores were at ceiling for both PLWH and HC. However, regarding UPSA-B total score, the mean value was significantly worse in PLWH when compared with HC [mean 82.1(SD 9.3) vs 89.2 (SD 6.2); *p* < 0.001] with a large effect size (*d* = 0.92). Exploring the UPSA-B subtests, mean communication score significantly differed between the two groups [mean 35.6 (SD 7.2) in PLWH vs 41.0 (SD 5.8) in HC; *p* = 0.002] with a large effect size (*d* = 0.83); although no significant difference was observed at financial skills [mean 46.5 (SD 4.6) in PLWH vs 48.2 (SD 2.4) in HC; *p* = 0.096], a medium effect size was found (*d* = 0.48).

In PLWH, the association between UPSA-B and cognitive performance (total score) was analyzed by linear regression analysis (see Table 3). At multivariate analysis, higher total cognitive Z-score was associated with better performance at UPSA-B total score after adjusting for education, smoking, and adherence.

**Table 3:** Factors associated to UPSA-B total score

	Univariate analysis			Multivariate analysis		
	Mean change	95% CI	*p*	Mean change	95% CI	*p*
Age (per 10-year increase)	− 0.62	− 0.25/0.12	0.519	–	–	–
Education (per 1-year increase)	0.71	0.16/1.27	**0.012**	0.18	− 0.32/0.69	0.463
Sex (male versus female)	1.86	− 2.97/6.69	0.445	–	–	–
Smoking	− 7.22	− 13.29/− 1.15	**0.020**	− 1.70	− 7.35/3.94	0.549
Cannabis use	− 10.93	− 24.05/2.18	0.101	–	–	–
Alcohol use	− 4.22	− 22.94/14.50	0.655	–	–	–
Other drug use	0.83	− 17.91/19.58	0.930	–	–	–
Years from HIV diagnosis (per 1-year increase)	0.009	− 0.09/0.11	0.869	–	–	–
Time from starting first cART regimen (per 1-year increase)	0.01	− 0.13/0.15	0.853	–	–	–
Time from starting current cART regimen (per 1-year increase)	0.01	− 0.07/0.19	0.724	–	–	–
Adherence (per 10% increase)	0.11	− 0.01/0.24	0.075	0.07	− 0.03/0.19	0.176
Past AIDS-defining events	1.43	− 3.05/5.90	0.527	–	–	–
CD4 cell count, cells/μL (per 100 cells/mL increase)	0.00	− 0.001/0.00	0.121	–	–	–
CD4 cell count nadir, cells/μL (per 100 cells/mL increase)	0.005	− 0.007/0.1	0.411	–	–	–
HIV RNA < 50 copies/mL	− 8.79	− 19.74/2.15	0.114	–	–	–
Injecting drug users	1.98	− 4.35/8.31	0.535	–	–	–
Hepatitis co-infection	− 5.50	− 14.2/3.1	0.211	–	–	–
Zung Depression Scale (per 1 point increase)	−1.11	− 0.38/0.15	0.389	–	–	–
Mean Z-score SNB (per 1 Z-score increase)	8.28	5.63/10.9	**< 0.001**	7.56	4.68/10.44	**< 0.001**

Bold values represent statistically significant *p* values

*CI* confidence interval, *cART* combined antiretroviral therapy, *SNB* Standardized Neuropsychological Battery test

Analyzing UPSA-B subtests, at univariate analysis, better cognitive performance (a + 1 Z-score increase in cognition is related to a 5.54 change in UPSA-B; 95% CI 3.39/7.74, *p* < 0.001) was associated with higher communication skills. At multivariate analysis, higher total cognitive Z-score confirmed the association (a + 1 Z-score increase in cognition is related to a 4.19 change in UPSA-B; 95% CI 1.80/6.59, *p* < 0.001) with better communication skills after adjusting for education, smoking, and cannabis use. Moreover, at univariate analysis, better cognitive performance (a + 1 Z-score increase in cognition is related to a 2.85 change in UPSA-B; 95% CI 1.38/4.33, *p* < 0.001) was correlated with higher financial skills; at multivariate analysis, higher total cognitive Z-score confirmed the association (a + 1 Z-score increase in cognition is related to a 2.77 change in UPSA-B; 95% CI 1.31/4.23, *p* < 0.001) with higher financial skills after adjusting for adherence.

Variables related to the severity of HIV infection (current and nadir CD4 cell count, time from HIV diagnosis, HCV co-infection, time from ART), mood status, and health-related quality of life were not found to be associated with EF performance measured by UPSA-B (both globally and in each subtest).

Furthermore, we investigated also the associations between each cognitive domain and UPSA-B scores. For simplicity, only significant associations at multivariate analyses where reported. Better memory score was associated to a higher performance at UPSA-B total (a + 1 Z-score increase in memory domain is related to a 3.38 change in UPSA-B; 95% CI 1.41/5.35; *p* = 0.001) and communication score (a + 1 Z-score increase in memory domain is related to a 2.20 change in UPSA-B; 95% CI 0.64/3.76; *p* = 0.006); higher language fluency was associated to better performance at UPSA-B total (a + 1 Z-score increase in language domain is related to a 2.28 change in UPSA-B; 95% CI 0.42/4.14; *p* = 0.012) and communication score (a + 1 Z-score increase in language domain is related to a 1.66 change in UPSA-B; 95% CI 0.26/3.07; *p* = 0.021); better attention performance was associated to higher values at UPSA-B total (a + 1 Z-score increase in attention domain is related to a 5.28 change in UPSA-B; 95% CI 2.93/7.62; *p* < 0.001) controlling for smoking, communication skills (a + 1 Z-score increase in attention domain is related to a 2.72 change in UPSA-B; 95% CI 0.82/4.63; *p* = 0.006) controlling for smoking, and financial score (a + 1 Z-score increase in attention domain is related to a 2.42 change in UPSA-B; 95% CI 1.24/3.60, *p* < 0.001); better executive functions were associated to higher values at UPSA-B total (a + 1 Z-score increase in executive functions domain is related to a 5.65 change in UPSA-B; 95% CI 2.49/8.80; *p* = 0.001) controlling for education and smoking, communication (a + 1 Z-score increase in executive functions domain is related to a 3.37 change in UPSA-B; 95% CI 0.92/5.81; *p* = 0.008) adjusting for education and smoking, and financial skills (a + 1 Z-score increase in executive functions domain is related to a 2.33 change in UPSA-B; 95% CI 0.66/4; *p* = 0.007); finally, higher motor abilities were associated to higher values at UPSA-B total (a + 1 Z-score increase in motor abilities domain is related to a 2.63 change in UPSA-B; 95% CI 0.88/4.39; *p* = 0.004) and financial score (a + 1 Z-score increase in motor abilities domain is related to a 1.48 change in UPSA-B; 95% CI 0.65/2.31; *p* < 0.001).

## Discussion

The aim of this study was to examine EF and its association with cognitive abilities among a sample of PLWH on cART with a very good immunological condition, by administering a new multi-domain and ecological assessment tool as the UPSA-B. The identification of a sensitive and reliable tool to assess EF is still an unresolved need in PLWH. Indeed, it is estimated that nearly 60% of PLWH presents problems with EF (Blackstone et al. 2013), probably due to a combination of several factors that are still not well understood. EF has also a central role in Frascati criteria classification since HAND subcategories are differentiated on the basis of absence/presence and severity of both cognitive and EF impairment (Antinori et al. 2007).

Our findings show that UPSA-B was much more sensitive than IADL, the most commonly used scale, to assess EF impairment in PLWH; indeed, our HIV-infected population, composed by patients with IADL scores at ceiling, performed significantly worse at UPSA-B when compared with HC. IADL has shown low sensitivity in patients with no overt dementia, partly related to the limitations of a self-reported questionnaire (Heaton et al. 2004); at the opposite, the UPSA-B has the advantage of considering both objective and self-report data in the determination of patients’ functional abilities, improving the capacity to detect also very mild EF impairment. According to IADL, all PLWH included in our study must be considered affected by ANI profile (Antinori et al. 2007); however, their performance at UPSA-B suggests that possibly some MND profile were undiagnosed.

Our results differed from other previous studies. Sheppard found that UPSA-B was ineffective to differentiate between HIV+ and HIV– patients (Sheppard et al. 2018), while Moore concluded that differences in UPSA-B performance could emerge when the HIV+ and HIV– groups are not matched for sex and education (Moore et al. 2017). In our study, the two groups where matched for education but not for sex; however, we found no association between sex and UPSA-B performance at univariate regression analysis. Considering the UPSA-B subtests (communication and financial skills), PLWH showed a significantly poorer performance at communication score when compared with HC. Similarly a lower performance at communication score was observed in another study, despite this observation showed only a trend towards statistically significance (Moore et al. 2017). A wider difference in our study could be explained by older age and lower education of our population when compared with the aforementioned study or by sociocultural difference in the Italian population. Anyway, our findings hint that PLWH could show a lower performance on tasks involving interactions with others, as suggested by Grabyan’s study (Grabyan et al. 2018) where PLWH with HAND were impaired on emotion identification task in association with a worse UPSA-B communication score.

Our findings suggested also a correlation between cognitive functioning and EF performance in PLWH. Indeed, we found an independent association between the performance at UPSA-B total score and the global cognitive performance. Moreover, higher performances at UPSA-B communication and financial subtests were independently associated to better global cognitive performance. However, we could not deeply explore if UPSA-B might help to differentiate PLWH participants with and without neurocognitive impairment (Sheppard et al. 2018) because all except 6 (7%) of our participants were cognitively unimpaired. Anyway, in countries where cART is widely available as Europe, up to 90% of PLWH on cART are successfully treated with stable virological suppression; in this context, the prevalence of HAND decreased to around 20% or less, with most patients showing the asymptomatic profile (Ciccarelli 2020; Fabbiani et al. 2017; Garvey et al. 2011; Milanini et al. 2016a, 2016b).

Furthermore, all cognitive domains assessed in the current study (memory, linguistic skills, attention values, executive functions, and motor abilities) were associated with UPSA-B total and communication scores, while UPSA-B financial score turned out associated with attention values, executive functions, and motor abilities. As suggested by Sheppard (Sheppard et al. 2018), the lack of a specific cognitive pattern related to UPSA-B performance might depend on the complex nature of everyday tasks performed in the real world.

Finally, variables related to the severity of HIV infection (current and nadir CD4 cell count, time from HIV diagnosis, HCV co-infection, time from cART initiation), mood status, and health-related quality of life were not associated with EF performance measured by UPSA-B.

We acknowledge that our study has some limitations. First of all, this is a cross-sectional study and uncontrolled biases could occur in routine clinical practice. Thus, future longitudinal studies are needed to confirm our findings and to better clarify the UPSA-B ability in measuring EF in the HIV+ population. Secondly, we investigated EF in a predominately male sample. There may be sex differences in the associations examined, and larger sample studies are needed to analyze them. Lastly our findings are applicable only to PLWH with a very good viroimmunological condition and with no neuroaids. Therefore, further investigations including more PLWH with HAND or with worse immunological conditions are needed, in order to identify also a good cut-point to discriminate abnormal UPSA-B performance. However, a well-controlled HIV-infected sample is more representative of the current Italian HIV-infected population.

In conclusion, UPSA-B seemed to better discriminate EF impairment than IADL in PLWH, and it was associated with cognitive functions, also in the absence of symptomatic cognitive impairment. On these bases, UPSA-B seems a promising tool to measure EF functioning in the context of HIV infection, and it could be useful to avoid misdiagnosis, to differentiate asymptomatic versus symptomatic HAND and to detect mild EF impairments in PLWH.
